# Incorporating Virtual Reality Agents During a Dichotic Speech Reception Task: Insights From the Heart

**DOI:** 10.1097/AUD.0000000000001597

**Published:** 2024-10-11

**Authors:** Bethany Plain, Hidde Pielage, Adriana A. Zekveld, Michael Richter, Tanveer A. Bhuiyan, Sjors R. B. van de Ven, Sophia E. Kramer

**Affiliations:** 1 Amsterdam University Medical Center, Vrije Universiteit Amsterdam, Otolaryngology Head and Neck Surgery, Ear & Hearing, Amsterdam Public Health Research Institute, Amsterdam, the Netherlands; 2 Eriksholm Research Centre, Snekkersten, Denmark; 3 School of Psychology, Faculty of Health, Liverpool John Moores University, Liverpool, United Kingdom; 4 Demant A/S, Kongebakken, Smørum, Denmark.

**Keywords:** Autonomic nervous system, Cardiovascular, Dichotic listening, Listening effort, Virtual reality

## Abstract

**Objectives:**

Listening effort is moderated by not only task difficulty, but also success importance. In real communication scenarios, success importance varies based upon the social context. However, in the laboratory, it can be challenging to manipulate social context without compromising experimental control. Outside of hearing sciences, studies have applied virtual reality (VR) to incorporate social context in a controlled and repeatable manner. Several of these studies have demonstrated that social manipulations in VR can reliably elicit changes in cardiovascular measures. Here, we investigated the effect of adding VR agents to a speech reception task, while measuring from the cardiovascular system.

**Design:**

Twenty-eight, normally hearing participants undertook a dichotic speech reception task. Sentences in stationary noise were presented dichotically, that is, different sentences presented simultaneously to each ear. Participants were tasked to either repeat one of the sentences (single-sentence condition) or both of the sentences (dual-sentence condition). The task was conducted under two VR conditions: (1) in the presence of agents, who provided sporadic performance feedback and (2) in the presence of nonagent controls, without any feedback given. Alongside task performance, we quantified changes in cardiovascular measures, relative to pretask baselines: heart rate variability, pre-ejection period, heart rate, and blood pressure. After each condition, participants rated their subjective effort, difficulty, performance, and engagement.

**Results:**

Performance and the subjective perception of performance were lower, while subjective effort and difficulty were higher, in the dual-sentence condition, compared with the single-sentence condition. Heart rate was the only cardiovascular measure that was sensitive to the experimental manipulations. Contrary to our expectations, heart rate increased in the nonagent control conditions, compared with the agent conditions. An exploratory analysis revealed heart rate fluctuations within a trial: heart rate was higher during the first 6 sec of the trial (reflecting the presentence masking noise and the sentence presentation) in the dual-sentence condition, compared with the single-sentence condition.

**Conclusions:**

This study was the first to incorporate VR agents who provided performance feedback during a dichotic speech reception task. Our results suggest that the VR agents did not increase success importance, which could be attributed to a lack of realism of the agents. We also demonstrated that the cardiovascular response to experimental manipulations may differ depending on the data window selected for analysis.

## INTRODUCTION

Listening effort is an increasingly investigated phenomenon, defined as “the deliberate allocation of mental resources to overcome obstacles in goal pursuit when carrying out a [listening] task” (Pichora-Fuller et al. 2016, p. 11S). In daily life, speech is commonly encountered in the presence of acoustic challenges (or obstacles), for example, interfering background noises, which must be ignored to selectively attend to the target talker (Smeds et al. 2015; Wu et al. 2018; Christensen et al. 2021; Shields et al. 2022). The process of directing attention in this manner can be demanding and can require substantial listening effort investment (Pichora-Fuller et al. 2016; Koelewijn et al. 2017). The increase in effort required when attention is divided between different speech sources has been demonstrated in several laboratory studies (Best et al. 2010; Koelewijn et al. 2014; Seeman & Sims 2015).

Another important aspect affecting listening effort in daily communication scenarios is the social context. Social context is thought to moderate “success importance”: the value that the listener places on understanding successfully (Matthen 2016; Pichora-Fuller 2016; Pichora-Fuller et al. 2016; Hughes et al. 2018; Shields et al. 2022). Considering the social contexts at play, a listener may determine whether the required effort is justified, and if not, they may disengage from listening (Brehm & Self 1989; Mackersie & Kearney 2017). When investigating listening effort in the laboratory, however, it is challenging to manipulate social context without compromising experimental control (Kvavilashvili & Ellis 2004). One way to maintain the desired level of experimental control and consistency is the application of virtual reality (VR) technology. In this study, we investigated the effect of incorporating VR agents during a dichotic speech reception task.

### Dichotic Listening

Attention is “a multidimensional construct that includes orienting, selecting, and/or focusing on environmental stimuli (e.g., speech) or internal representations (e.g., thoughts)” (Pichora-Fuller et al. 2016, p. 11S). Several studies have investigated the effects of dichotic listening in the laboratory (Best et al. 2006, 2010; Shinn-Cunningham & Best 2008; Koelewijn et al. 2014). For example, Best et al. (2010) presented simultaneous, independent streams of masked speech dichotically to normal-hearing listeners. Participants undertook two different task conditions: a single-attention condition, where they had to attend to and repeat key words presented to one ear, while ignoring stimuli simultaneously presented to the other ear, or a dual-attention condition, where they had to attend to and repeat key words presented simultaneously to both ears. In the dual-attention condition, the accuracy at which participants could repeat target words deteriorated, compared with the single-attention condition (Best et al. 2010).

Physiological measures have been used during dichotic listening tasks, as a proxy of listening effort (Koelewijn et al. 2014; Seeman & Sims 2015). For example, Koelewijn et al. (2014) conducted a similar study design to that of Best et al. (2010), with the addition of pupil dilation measures. They reported that the dual-attention condition elicited increased cognitive processing load (demonstrated by a larger pupil dilation) as compared with the single-attention condition. Rather than pupil measures, Seeman and Sims (2015) incorporated cardiovascular measures into their dichotic listening task. Their participants repeated digits in three different configurations, increasing in task complexity: diotic single digit (one digit to repeat), dichotic single digits (two digits to repeat, one from each ear) and dichotic double digits (four digits to repeat, two from each ear). The results demonstrated a significantly larger change in heart rate variability (HRV) relative to baseline during the two dichotic conditions, compared with the diotic condition, suggesting increased effort investment (Seeman & Sims 2015).

### Social Context and VR

The social context of the communication scenario is also important in determining listening effort investment (Matthen 2016; Pichora-Fuller 2016; Hughes et al. 2018). Despite this knowledge, laboratory research into listening effort has rarely implemented social context manipulations. To our knowledge, just five studies have investigated social context during speech-in-noise tasks, each applying a different paradigm: (1) participants answered a set of questions evaluating the level of their comprehension of the speech material they heard (Picou & Ricketts 2014), (2) participants were told that video footage of them performing the task would be later evaluated by a panel of experts (Mackersie & Kearney 2017), (3) pairs of participants performed the task in tandem (Pielage et al. 2021), (4) two strangers passively observed participants during a listening task (Plain et al. 2021; Pielage et al. 2023), and (5) the experimenter provided explicit performance feedback and encouragement to “please try harder” during the task (Zekveld et al. 2019). The evaluative manipulations in these studies aimed to draw upon social-evaluative threat, or the fear of being negatively judged by others, which is commonly experienced in daily life (Dickerson & Kemeny 2004; Jonas & Mühlberger 2017).

These five paradigms varied in their level of experimental control and strength of evaluation, as well as their degree of ecological validity (Keidser et al. 2020). The evaluative aspect of the first two studies was achieved by means of the instructions provided to participants. In their evaluated condition, Picou and Ricketts’ (2014) participants were told that their comprehension of the sentence material would later be evaluated by means of a set of questions. In Mackersie and Kearney (2017) study, participants were told they were being video recorded and that a panel of experts would later review the footage. While providing evaluation by such means is well-controlled and highly repeatable, studies investigating cortisol responses to social-evaluative threat have revealed that video recording-based methods elicit smaller responses than evaluation by physically present evaluative others (Dickerson & Kemeny 2004). It is likely that real-time evaluation and the physical presence of an evaluator are more relevant to the participant than video footage of them being reviewed later.

The evaluative aspect of the other three studies included the physical presence of one or more human interlocutors, with the designs varying depending on whether these individuals were untrained or trained. Two of the studies (2019) included nontrained individuals in their designs. Pielage et al. (2021) included two participants in the same room simultaneously, who both performed speech perception testing by alternating sentence repetition during the task. In contrast, Plain et al. and Pielage et al. (2023) included two strangers who were seated within the participant’s eyeline and passively observed and evaluated them performing the task. It could be argued that these two experiments were susceptible to variability and lacked experimental control, because people are inherently unpredictable in their behavior. For example, even though interactions between the individuals were minimal, each participant pair or trio will have had a slightly different social dynamic. Such social interactions are difficult to control, quantify, and replicate.

These limitations affecting experimental control can be alleviated by the inclusion of evaluators trained using a standardized protocol. Zekveld et al.’s (2019) participants were explicitly urged by the experimenter—a trained individual—to try harder to improve their performance to reach an impossible target performance level. Here, the evaluation was repeatable and standardized across study participants. Outside of hearing sciences, the impact of trained evaluators is well documented, particularly in the literature regarding the Trier Social Stress Test (TSST). The TSST involves participants undertaking challenging tasks, including public speaking and mental arithmetic, in the presence of a physical audience (Dickerson & Kemeny 2004). This evokes physiological responses, including neuroendocrine and cardiovascular responses, interpreted to reflect social-evaluative threat (Allen et al. 2014). However, it has been noted that the TSST procedure may vary slightly between sites due to different laboratory environments and different audiences (Jönsson et al. 2010). In addition, it is resource-heavy due to the requirement for trained evaluators (Shiban et al. 2016).

A promising way in which to simulate social scenarios in the laboratory, while maintaining experimental control and repeatability, is the development of VR test scenarios (Kothgassner & Felnhofer 2020). The impact of a VR TSST has been investigated and crucially, VR adaptations of the TSST (i.e., the audience members are agents rather than physical people), have been shown to elicit similar, albeit in some cases smaller, physiological responses to the real-life TSST (Zimmer et al. 2019; Fallon et al. 2021). To our knowledge, no previous listening effort study has included agents in VR to provide performance evaluation.

### Cardiovascular Measures During Listening Tasks

During listening tasks, physiological responses have been reported as correlates of listening effort, including changes to pupil size, skin conductance, and cardiovascular measures (McGarrigle et al. 2014; Pichora-Fuller et al. 2016; Zekveld et al. 2018). Though useful indicators, it should be noted that the physiological measures are unspecific: alone, they do not enable distinction between different cognitive processes (Zekveld et al. 2018; Richter et al. 2023). For example, effort and emotion may result in similar physiological responses (Francis & Love 2020). Of the aforementioned measures, cardiovascular measures are relatively unique as they allow for simultaneous and distinct assessment of activity from both branches of the autonomic nervous system: the sympathetic nervous system, SNS, and the parasympathetic nervous system, PNS (Berntson et al. 1991; Lovallo 2005; McCorry 2007). For example, pre-ejection period (PEP), the interval between the onset of excitation of the left ventricle and the opening of the aortic valve, is predominantly a measure of SNS activity (Ahmed et al. 1972; Newlin & Levenson 1979), whereas HRV, representing the fluctuation of the interval between heart beats, is considered a measure of PNS activity, depending on the metric used (Shaffer & Ginsberg 2017). Other relevant psychophysiological measures including heart rate and blood pressure are controlled by mixed autonomic origins (Gordan et al. 2015; Plain et al. 2021).

Due to their autonomic origins, cardiovascular measures hold promise in demonstrating physiological changes associated with effort. Despite this, the current picture of cardiovascular reactivity during listening is somewhat unclear. During speech reception tasks, HRV has been demonstrated to be sensitive to changes in talker rate (Mackersie & Calderon-Moultrie 2016) and signal to noise ratio (SNR) (Mackersie et al. 2015; Seeman & Sims 2015), but similar effects were notably absent in other studies (Mackersie et al. 2015; Plain et al. 2021). PEP has also been shown to be sensitive to SNR in some studies (Plain et al. 2020; Slade et al. 2021), but not in others (Plain et al. 2021).

One possible reason for these inconsistent results may relate to the data window used for the analysis. In general, studies have averaged measures across a whole task block, including data collected during masking noise presentation, sentence presentation, the participant verbal response, and experimenter scoring time (Mackersie et al. 2015; Seeman & Sims 2015; Mackersie & Calderon-Moultrie 2016; Plain et al. 2020, 2021). It is possible that by doing so, some of the dynamics of transient responses at the trial level are overlooked. Supporting this, a recent study demonstrated a more sensitive PEP response to SNR when the data corresponding to the stimulus presentation interval were analyzed only, as opposed to all data within the block (Plain et al. 2020). Furthermore, Francis et al. (2016) demonstrated that heart rate fluctuated during trials of a speech reception task. They showed that compared with a pretrial baseline, heart rate decreased at around 6 sec after trial onset. More work is needed to elucidate the trial-level dynamics of cardiovascular measures.

### Aims and Hypotheses

The present study manipulated task demand during a dichotic listening task, while incorporating agents or nonagent controls (stacked boxes displaying small idle movements; see more detail in VR manipulation: agents and nonagent controls) into the virtual environment. The task consisted of two dichotic listening conditions: single- and dual-sentence. In both, speech stimuli were presented dichotically (i.e., different sentences presented to each ear in stationary noise). In the single-sentence condition, participants repeated the sentence presented to one ear, ignoring the sentence in the other ear, whereas in the dual-sentence condition, they repeated both sentences, one presented to each ear. We manipulated success importance by adding agents or nonagent controls into the virtual environment: participants performed the task in the presence of two agents who provided occasional nonverbal performance feedback (head nod for correct answers, head shake for incorrect answers), or in front of nonagent visual controls without feedback. We measured sentence repetition performance, self-reported measures of effort, task difficulty, performance and engagement, and physiological responses from the cardiovascular system and pupils of participants (the pupil data will be presented elsewhere).

We hypothesized that sentence repetition performance (proportion of words from both sentences) would decrease in the dual-sentence condition compared with the single-sentence condition, as demonstrated in similar studies (Best et al. 2010; Koelewijn et al. 2014). We expected that in the single-sentence condition, the presence of agents and feedback would increase success importance, motivating participants to invest more effort compared with the nonagent control condition. This would be reflected by the self-report measures and indexed by a shortening of PEP, a decrease in high-frequency HRV (HF-HRV) and an increase in heart rate. In the dual-sentence condition, we expected an overall increase in effort investment compared with the single-sentence condition, because the task was more challenging. We also anticipated that the effect of the virtual agents would be diminished in the dual-sentence condition because the cognitive load of dividing attention would override the effect of the agents. That is, the dual-sentence condition may be demanding enough to elicit a ceiling effect, such that the presence of the agents would have no measurable additional impact.

An additional aim of the study was to explore the timings of cardiovascular changes within speech reception task trials. There is evidence to suggest that changes to heart rate occur relatively quickly, that is, within seconds, in response to sound (Francis et al. 2016; Shoushtarian et al. 2019). Furthermore, as described earlier, a previous study revealed higher sensitivity of PEP reactivity when only the listening components were analyzed (Plain et al. 2020). By averaging across full blocks of sentences it is possible that physiological changes are missed. In this additional exploration, we tested the same hypotheses but instead selected three parts of the trial to analyze the physiological responses in the following intervals: during the masking noise preceding the sentence, during sentence presentation, and finally, during the postsentence masking noise.

## MATERIALS AND METHODS

### Participants

The study was advertised by means of distribution of flyers, both physically at university buildings and via Facebook posts shared in local groups. To determine the sample size, a power calculation was performed upon previous work using HRV (0.74 η_p_²) and heart rate (0.25 η_p_²) during different task complexity levels of a dichotic digit repetition task (Seeman & Sims 2015). Our power calculation, completed in G*Power 3.1.9.4 software, referred to a repeated measures analysis of variance (ANOVA) with an estimated univariate effect size of 0.25, an alpha error of 0.05, power of 0.8, and a correlation of 0.5 between repeated measures. This calculation indicated that 24 participants were required. Four more were included, as we anticipated a high possibility of data exclusion when preprocessing the physiological measures.

First, 12 pilot participants were recruited and tested with a shortened experimental protocol without baselines. Subsequently, 28 participants underwent the full experimental protocol—results from these participants are reported here. Two of these had to be excluded because of cardiovascular data quality issues, therefore data from the remaining 26 participants (11 males, 15 females) will be presented here. Participants were native Dutch speakers, right-handed, and normally hearing (≤20 dB HL at 0.5, 1, 2, and 4 kHz), with a mean age of 26.5 years (SD = 4.4 years). They reported no history of psychiatric, neurological, ocular, or cardiovascular problems. Participants with long or short-sightedness were encouraged to wear lenses, rather than glasses, during the experiment. All participants provided informed consent in accordance with local ethical committee procedures.

### Procedure and Apparatus

Testing was conducted in a sound-treated room. Participants were seated on a centrally placed chair, in front of a table. The experimenter controlled the equipment from an adjacent room, outside the participant’s view. During the experiment, participants wore an HTC Vive pro VR headset with headphone attachments, which were used to present all audio stimuli. The virtual environment was designed and implemented in 3D game engine software, unity, using additional SteamVR software. Custom-made C# scripts were written to run the experimental protocol.

Participants attended a single test session lasting around 2 hr in duration. At the start of the test session, the experimenter explained all procedures and obtained the participant’s written informed consent to proceed. Following this, pure tone audiometry was conducted at 0.5, 1, 2, 4, and 8 kHz, to ensure that participants met the audiometric inclusion criteria. Next, participants had their height and weight measured by the experimenter, such that body mass index (BMI) could be calculated. Then, the experimenter applied the cardiovascular electrodes and blood pressure cuff. After a thorough explanation of the task, the VR headset was applied to the participant and calibration of the eye tracker within the VR headset was conducted.

Participants were then given the opportunity to acclimate to the VR environment, while they practiced the task: four practice trials were presented, two single-sentence trials, and two dual-sentence trials. During the practice, participants were also familiarized with the agents, nonagent controls, and the animations of both. Aside from moving their head and observing the animated objects, participants could not interact with the VR environment. Subsequently, the four task blocks were conducted. Each task block contained 30 sentences and lasted around 10 min in total. The order of task conditions was counterbalanced between participants, resulting in 24 unique order combinations with two repeated orders.* Each block was preceded by a 3-min baseline video and ended with the participants removing the headset to complete subjective rating scales on paper. Electrocardiography (ECG) and impedance cardiography (ICG) were measured throughout the experiment. The blood pressure cuff was inflated once in the middle of each baseline and once during each task block. Pupil size was also recorded during the task blocks. The pupil data will be reported elsewhere (Pielage et al. n.d.). Breaks were offered to participants after two blocks. When the four task blocks had been completed, participants completed two questionnaires: the i-group presence questionnaire (Schubert et al. 2001) and a questionnaire about the agents. Last, participants were debriefed and the procedure for reimbursement was discussed.

### Dichotic Listening Test Blocks

The experiment consisted of a two (dichotic speech reception task demand) by two (VR manipulation) within-subject design.

#### Dichotic Speech Reception Task Demand

The dichotic speech reception task was inspired by previous work (Shinn-Cunningham & Best 2008; Best et al. 2010; Koelewijn et al. 2014). Per task block, 30 Dutch, everyday sentences were presented dichotically in speech-shaped stationary noise, the level of which was 65 dB SPL (Versfeld et al. 2000). The stationary noise, referred to here as masking noise, preceded each sentence by 3 sec and continued for 3 sec after sentence offset. Sentence presentation lasted on average 1.84 sec but ranged from 1.3 to 2.7 sec. The SNR remained fixed throughout at −3 dB. The target sentence presented to the left ear was spoken by a female talker, and the target sentence that was simultaneously presented to the right ear was spoken by a male talker. The number of words in the sentences varied, with on average of six words per sentence (Versfeld et al. 2000).

After the offset of the masking noise, participants were tasked to (1) attend to the sentence spoken by the female (left) and ignore the male talker (right), referred to as the single-sentence condition, or (2) to attend to both speakers simultaneously, referred to as the dual-sentence condition. Thus, in the single-sentence conditions they had to repeat one sentence, but in the dual-sentence conditions they had to repeat two sentences, one from the left ear followed by one from the right ear. Information about which ear to attend to was given to participants verbally before the task block, with no additional auditory or visual cues provided during the task. Participants were encouraged to guess if they were not confident about their answers. Scoring was conducted live during the experiment by an experimenter who was seated outside the sound-treated room. The scoring was based upon all words in the target sentences. Errors in word repetition were not permitted (for instance incorrect tenses, articles, or singular/plural errors), however, errors in word order were treated more leniently (correct words repeated in the wrong order were scored as correct). The scoring was recorded as a proportion of words correctly repeated, out of the total target words in the sentence. There was one target sentence per trial in the single-sentence condition and two target sentences per trial in the dual-sentence condition. There were therefore approximately twice as many words to recall as in the dual-sentence condition as compared with the single-sentence condition.

#### VR Manipulation: Agents and Nonagent Controls

**Fig. 1.**
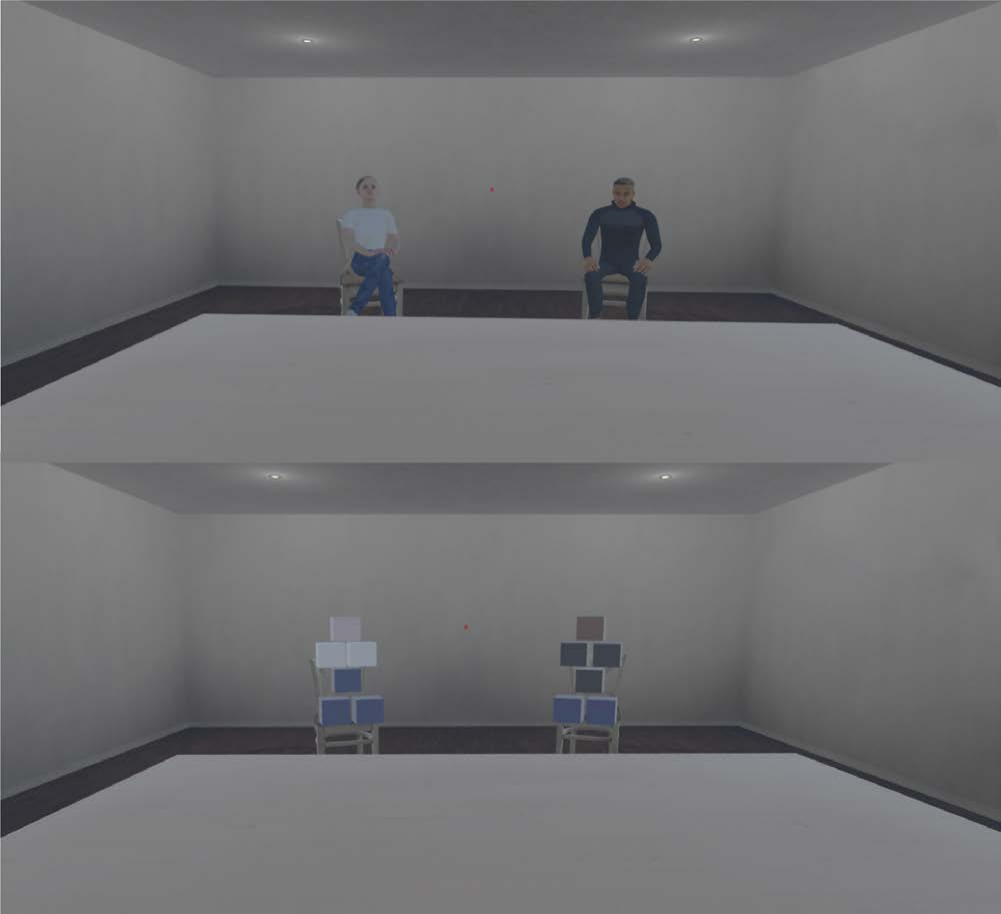
Screenshots of the virtual reality environment visible to the participant. The upper panel demonstrates the two agents who provided occasional performance feedback, whereas the lower panel demonstrates the nonagent controls who gave no feedback. All other aspects of the room were identical in both the agent and nonagent control conditions.

The virtual scene was manipulated by the introduction of agents or nonagent controls. Within the headset, the virtual environment was 9 meters by 6 meters wide and consistent with the physical laboratory environment, contained a centrally placed chair and a table. Unlike the physical laboratory, the virtual scene contained two additional chairs, positioned 4.5 meters away from the participant’s chair, and each 1 meter to the left or right. There were also two windows (out of the view of participants when gazing forward) and a permanent red dot on the back wall, upon which participants were told to direct their gaze during sentence presentation. The participant had no body in the virtual environment. Participants undertook the task in two conditions: (1) in the presence of two agents who gave occasional performance feedback or (2) in the presence of two nonagent controls. Figure 1 shows the virtual environment in both of these conditions.

The agents’ physical features were downloaded from Mixamo (https://www.mixamo.com, Mixamo characters selected: David and Megan). The characters, one male and one female, were selected based upon their level of human resemblance, in addition to their age demographic and casual clothing which could reasonably place them as peers of our participants. Both agents were seated on chairs in the virtual scene, within the participant’s eyeline. They displayed looped, somewhat natural-looking idle behavior, such as breathing and small movements, rather than being completely still. When present, the agents occasionally provided performance feedback to the participant. Feedback lasted for 2 sec in duration. Positive feedback consisted of an animation of the agent/s nodding their head, and negative feedback consisted of an animation of the agent/s shaking their heads. In a trial, participants could receive feedback from either the male agent, the female agent, or both together. Opposing feedback was never given by the agents (i.e., simultaneously positive and negative feedback). To receive positive feedback, participants had to successfully repeat a full sentence during the single-sentence condition, or two words of each sentence during the dual-sentence condition. If these criteria were not met, negative feedback could be given.

The frequency of feedback was matched approximately equally between the two dichotic conditions, as well as between participants. In general, the chance of feedback being provided by one of the agents in a trial was 15%. However, to account for the increased likelihood of participants earning positive feedback in the single-sentence condition, the chance of negative feedback being given was increased to 80%, relative to positive feedback during that condition. To ensure a similar amount of feedback between conditions, the agents were each programmed to show a maximum of three negative and five positive feedback animations per block. As expected, there was a significant effect of feedback type (positive versus negative) on the number of feedback responses given (i.e., the sum of both agents’ responses), such that there were more positive responses given than negative [*F*(1,25) = 115.54, *p* ≤ 0.001, *η*_p_² = 0.82]. In addition, an interaction effect was present [*F*(1,25) = 23.07, *p* ≤ 0.001, *η*_p_² = 0.48], suggesting that the difference between positive and negative feedback was more pronounced in the single-sentence, rather than dual-sentence condition. This is likely to result from the higher performance in this condition than in the dual-sentence condition—there were fewer opportunities for negative feedback to be given. There was no significant difference between the amount of negative and positive feedback given by the male compared with the female agent [in dual-sentence condition, *F*(1,25) = 2.44, *p* = 0.13, *η*_p_² = 0.09; in single-sentence condition, *F*(1,25) = 0.72, *p* = 0.40, *η*_p_² = 0.03].

The nonagent controls, on the other hand, consisted of boxes matching a similar color scheme to the agents. They also displayed idle behavior (moving a little, to match the agent idle movements) but did not provide any performance feedback. These nonagent controls were introduced such that we could differentiate whether any effects were resulting from the presence of an object in the visual field, versus an effect of the agent appearance plus feedback.

### Baseline Videos

Three-minute baselines were implemented before each task block, such that reactivity (delta) scores could be calculated for the cardiovascular measures. The purpose of these periods was to allow participants to enter a resting state (Jennings et al. 1992). During these baseline periods, participants watched videos projected onto part of the back wall of the virtual environment. The agents and nonagent controls were present during the baselines preceding the tasks blocks. The videos consisted of footage that was shown for the baselines of a previous experiment (Plain et al. 2021). The videos lasted for 3 min and depicted drone footage shot over Edinburgh’s countryside and cityscape. They were neutral in nature, containing no emotive, distressing, or stressful content.

### Subjective Ratings and Questionnaires

After each block, the head-mounted display was removed, and participants completed four separate rating scales about the task using pen and paper. They were asked to rate the following, “How much effort did it take you (on average) to understand the speech?,” “How difficult did you find the task?,” “Did you understand very few or almost all sentences?,” and “To what extent did you feel included in (engaged with) the experiment?.” To answer, participants selected their response on a paper visual analog scale that ranged from 1 to 10, with one decimal precision. Each scale had five written anchors, of which the extremes were labeled as follows: “no effort” to “very much effort,” “not at all difficult” to “very difficult,” “no sentences understood” to “all sentences understood,” and finally, “not included at all” to “fully included.” After that, the head-mounted display was adjusted again.

After the experiment, participants also completed two questionnaires on paper: the i-group presence questionnaire (Schubert et al. 2001) and a custom-made questionnaire, referred to here as the agent questionnaire. The i-group presence questionnaire measured the user’s subjective sense of presence during the experiment. Fourteen items were presented, each consisting of a statement about general presence, spatial presence, involvement, and experienced realism. For example, “I was completely captivated by the virtual world.” The response format consisted of a five-point Likert scale with labeled anchors at the extremes only (e.g., fully disagree to fully agree).

The agent questionnaire aimed to provide more information about the perception of the agents themselves. Five items were presented on a seven-point Likert scale with the labeled extremes presented in brackets here: (1) I was aware of the presence of the agents (strongly agree to strongly disagree); (2) it was like the agents were real (strongly agree to strongly disagree); (3) because of the presence of the agents I felt (less to more) relaxed; (4) because of the presence of the agents I felt (encouraged to discouraged); and (5) compared with when the agents were absent, when they were there I tried (less to more). Additional answer space was provided so that participants could elaborate on their answers if necessary.

### Cardiovascular Data Collection

Cardiovascular data were collected throughout the experiment by a Cardioscreen 2000 system (Medis, Ilmenau, Germany). The system measured ECG and ICG, both at a sampling frequency of 1000 Hz. These signals were obtained by three disposable electrodes positioned on the participant’s neck and chest. More specifically, a dual sensor was placed on the left side of the neck, and two single sensors were placed on the left side of the chest: one at the level of the xiphoid process and one 10 cm beneath. Participants wore a blood pressure cuff on their right arm, placed over the brachial artery. The blood pressure cuff was inflated once per baseline and once per task block (at around the 24th sentence), with around 6 min between successive inflations. The inflation of the blood pressure cuff lasted on average of 30 sec.

### Cardiovascular Measures

For the purposes of our analysis, we extracted cardiovascular measures using two different methods. In the first approach, referred to here as the block-wise method, the measures were calculated using an entire task block of data, averaged, and compared with the baseline values. Using this method, we extracted HRV, PEP, heart rate, and blood pressure, generating one reactivity score per condition. The included measures and the direction of expected effort-related effects can be seen in Table 1. This is the more “traditional” approach to cardiovascular psychophysiological measures. However, some studies have suggested that there are fluctuations in cardiovascular measures occurring within a trial (Francis et al. 2016), which may be missed when averaging across the whole block. Therefore, in the second approach, referred to here as the trial level method, we explored the measures when extracted instead at the level of the trial, averaged across 1-sec epochs. Such a short data window precludes HRV and blood pressure measures, which require a longer window of data. Therefore, using this trial-level method we only extracted changes in heart rate and PEP.

**TABLE 1.** Included cardiovascular measures, abbreviations, and the expected effort-related change

Measure	Abbreviation	Expected Effort-Related Change
High-frequency heart rate variability	HF-HRV	Decrease
Pre-ejection period	PEP	Decrease
Heart rate	HR	Increase
Systolic blood pressure	SBP	Increase
Diastolic blood pressure	DBP	Increase
Mean arterial pressure	MAP	Increase

#### Block-Wise Analysis

##### Heart Rate Variability

HF-HRV and heart rate were both extracted from the ECG signal. Full baseline and task data were imported to MATLAB (version R2018b). Any segments containing visible artifacts were removed, such that all included data were free from noise. A peak detection function was used to find all R peaks in the signal. Visual inspection of the R peaks demonstrated that the appropriate threshold had been selected and all peaks had been properly detected. Next, the interbeat intervals were loaded into Kubios HRV Standard 3.3.1 (Tarvainen et al. 2014), with artifact correction set to low (threshold: 0.3). Normalized HF-HRV, which is normalized using the ratio of the power in the frequency bands 0.15 to 0.4 Hz (high) and 0.04 to 0.15 (low), was extracted. This resulted in one HF-HRV value per task block and one per baseline. Delta reactivity scores were calculated for HRV by subtracting the baseline from the task values.

##### Pre-Ejection Period

PEP was extracted from the ECG and ICG signals. First, R peaks were detected in the ECG signal and visual inspection confirmed that detection was appropriate. The ICG signal was differentiated, and a low-pass Butterworth filter (order four, cutoff 50 Hz) applied. All individual cycles of the ECG and ICG signals were inspected, and any cycles containing artifacts were excluded. The remaining, artifact-free cycles were ensemble averaged across segments of 60 sec. The ensemble-averaged data were used to find PEP, which refers to the time interval between the R-onset of the ECG signal and the B-point of the ICG (selected using the procedure described by Sherwood et al. [1990]). This procedure resulted in three PEP values per baseline (which lasted 3 min) and from 8 to 14 PEP values per task block (the task duration was unfixed and depended upon the speed at which the participant completed sentence repetition).

To ensure reliability, scoring of PEP was conducted by two separate scorers. The PEP values from both scorers were compared with one another. Any differences greater than 10 msec were reviewed in a meeting between the scorers, and any errors in scoring were corrected, as necessary. The level of agreement between the two scorers was high: the intraclass correlation coefficient (two-way mixed, absolute agreement) was 0.98 before the scorers’ meeting and 0.99 after errors had been corrected. Final PEP values were calculated as an average of the two scorers’ PEP scores. PEP was averaged across the full 3-min baseline period and from minutes one to eight of the task period. This time period was selected for the task to ensure that all participants’ PEP score contained an average of the same number of minutes, because participants completed the task blocks at different times. The shortest task period was 8 min in duration. Lastly, delta reactivity scores were calculated by subtracting the average baseline PEP from the average task PEP.

##### Heart Rate and Blood Pressure

Heart rate was determined from the interbeat intervals obtained during HF-HRV extraction (Heart rate variability). The interbeat intervals were converted to heart rate (in beats per minute) and mean heart rate was calculated for each condition. Three individual blood pressure measures were extracted per condition: mean arterial pressure (MAP), systolic blood pressure (SBP), and diastolic blood pressure (DBP). Delta reactivity scores for heart rate and blood pressure were calculated by subtracting baseline values from the task values. Trials coinciding with blood pressure cuff inflation were included in the analysis.

#### Exploratory Trial Level Analysis

**Fig. 2.**
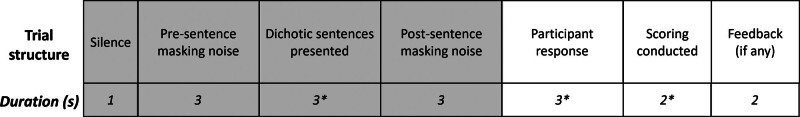
Schematic demonstrating the trial structure. The shaded section (consisting of the silence, presentence masking noise, dichotic sentence presentation, and postsentence masking noise) reveals the 10-sec window selected for the exploratory analysis. Trial durations are given in seconds. Those marked with an asterisk are approximate timings, as these sections of the trial had an unfixed duration.

Two participants were excluded from the exploratory trial level analysis due to an issue with triggering that compromised detection of their data corresponding to individual trials. Therefore, 24 participants were included in the analysis of heart rate and 22 participants were included in the analysis of PEP. Using MATLAB, ECG, and ICG data were filtered using sixth-order Butterworth band-pass filters with a passband of 1 to 30 Hz (Raza et al. 1992). The data corresponding to each trial were extracted from 1 sec before trial onset until 9 sec after trial onset. This 10-sec analysis window spanned from 1 sec before the presentence masking noise to the approximate end of the postsentence masking noise (shaded in Fig. 2). Data after this (i.e., during the response time, scoring, and any feedback presentation) were not included in the exploratory cardiovascular analysis.

After the 1 sec of silence, the trials were split into three sections, based upon the approximate acoustic sections of the trial. The first 3 sec always corresponded to the masking noise presentation alone. This section will be referred to as presentence masking noise. Sentence presentation varied from 1.3 to 2.7 sec in duration. To ensure all the sentences (and any instantaneous physiological changes elicited by sentence presentation) were adequately captured in our time window, we considered the next 3 sec to correspond to the dichotic sentence presentation. Last, the remaining 3 sec were considered to represent the postsentence masking noise. We acknowledge that these latter two-time windows are approximate and may be contaminated with other trial sections, however splitting the data in this way allowed us to compare timings across trials with different durations.

The data obtained during each trial were split into 1-sec epochs. For each epoch, the R peak of the ECG was detected using the same peak detection function referred to in Heart rate variability. Individual scripts were created to allow customized peak detection thresholds, and visual inspection of the signal was carried out to ensure the correct threshold had been chosen. When the correct threshold had been determined, the interbeat interval between this R peak and the subsequent R peak was calculated. If two or more R peaks were present in the epoch, the average of the interbeat intervals was calculated. In this way, one interbeat interval was obtained per one-second epoch. Interbeat intervals were then mathematically converted to heart rate and the values were normalized to the heart rate value calculated during the silence preceding the trial.

The same epoch-based process was conducted for PEP. In contrast with the approach described in Pre-ejection period, trial-level PEP was not obtained by manual scoring. Instead, a semiautomatic procedure was devised and implemented in MATLAB to detect the Q point of the ECG using peak detection, and the B-point of the ICG using a tangential method. Visual inspection was conducted to ensure adequate positioning of these points. The distributions of the heart rate and PEP values obtained from each participant were reviewed, and any outliers that were considered to be physiologically unlikely were excluded. Values were averaged across each condition (30 trials), such that each participant had nine average heart rate and PEP values, representing the proportional change from pretrial value for each second of the trial.

For both heart rate and PEP, the areas under the curve were calculated in MATLAB using trapezoidal numerical integration. Calculating area under the curve can allow statistical analysis of discrete-time course data (Jaki & Wolfsegger 2009). The advantage of doing so here was that the distinct parts of the trial could be analyzed without averaging across time and potentially losing important information. An increase in the area under the curve was interpreted to reflect an increase in heart rate or PEP, respectively.

### Statistical Analysis

Statistical analysis was conducted in IBM SPSS statistics (Version 28). Two-way repeated measures ANOVAs were conducted to determine effects (main or interaction) of the task demand condition (single- or dual-sentence) and the VR manipulation (agents or nonagent controls) on performance, cardiovascular reactivity measures, self-reported effort, difficulty, performance, and engagement. The area under the curves of the three trial sections (presentence masking, dichotic sentence presentation, and postsentence masking) was also analyzed by repeated measures ANOVAs. To account for the inclusion of multiple cardiovascular measures in the block-wise analysis and three different time points in the trial-level analysis, all cardiovascular *p* values were corrected for multiple comparisons using the false discovery rate Benjamini–Hochberg correction (Benjamini & Hochberg 1995; Bird & Hadzi-Pavlovic 2014; Martínez-Cagigal 2021).

## RESULTS

### Performance

**Fig. 3.**
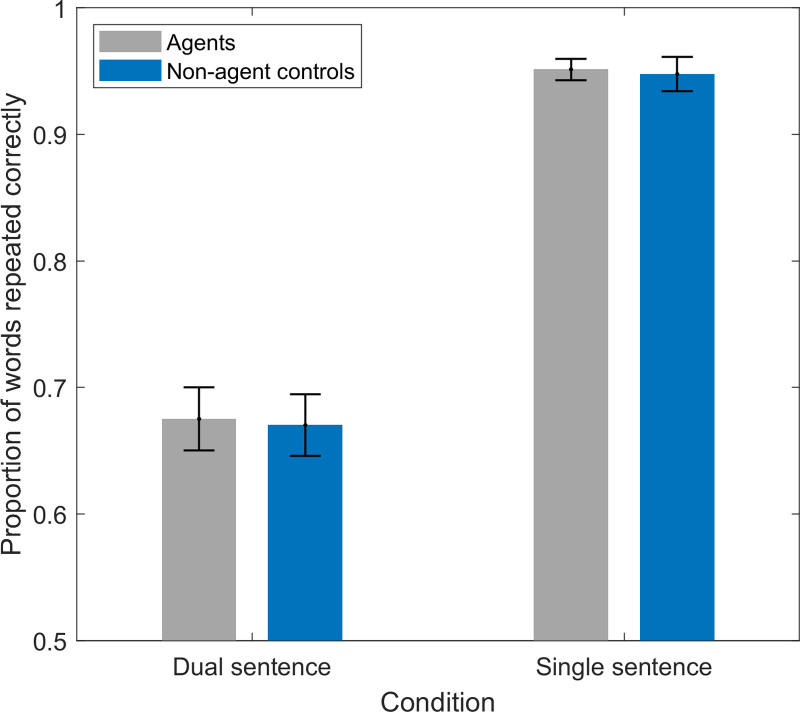
The average proportion of words repeated correctly in the dual- and single-sentence conditions, in the presence of the agents and nonagent controls. Error bars represent SE of the mean.

**Fig. 4.**
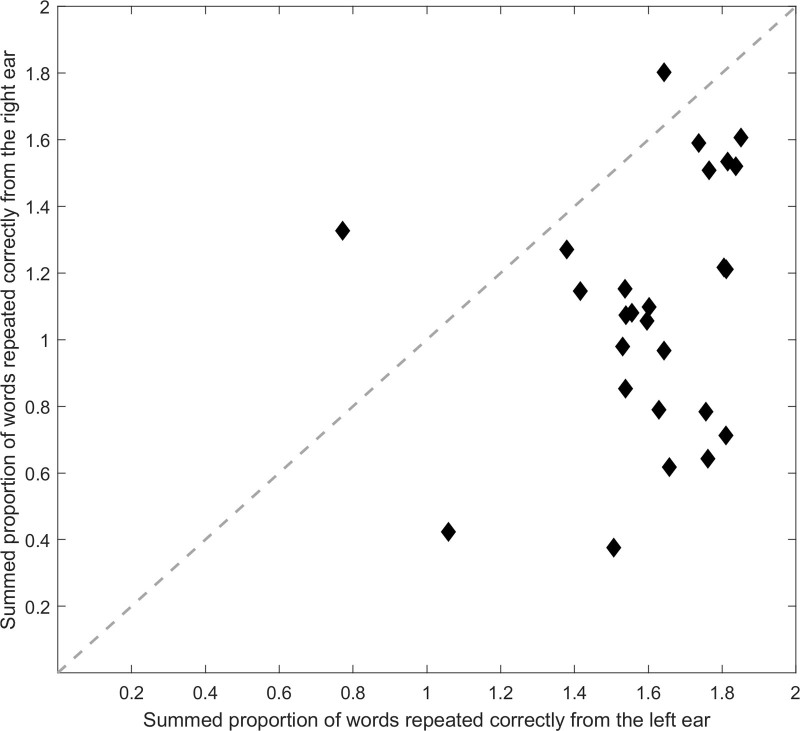
A scatter plot of the summed average proportion of words repeated correctly from the left ear compared with the right ear. The proportion of words in the agent and nonagent control conditions are added together to make a single score per participant. Each diamond represents one participant.

The total number of words correctly repeated in each condition is reported in Table 2. For statistical analysis, the proportion of words correctly repeated was calculated by dividing the sum of the correct words by the total number of presented words for each trial (there were twice as many target words for the dual-sentence condition compared with the single-sentence condition). The average proportional performance in each condition is presented in Table 2 and Figure 3. A two-way repeated measures ANOVA was conducted to assess the effects of task demand condition and VR manipulation on proportional performance. The results revealed a significant effect of task demand [*F*(1,25) = 163.55, *p* < 0.001, *η*_p_² = 0.87], such that proportional performance was higher in the single-sentence condition compared with the dual-sentence condition. There was no main effect of the VR manipulation [*F*(1,25) = 0.22, *p* = 0.64, *η*_p_² = 0.01] and no interaction between task demand and the VR manipulation [*F*(1,25) = 0.01, *p* = 0.93, *η*_p_² = 0.00]. A scatter plot demonstrating the summed proportion of words repeated correctly from each speaker in the dual-sentence condition indicated that the participants preferentially repeated words presented to the left ear as compared with the right ear (Fig. 4). This observation is consistent with previous studies that implemented a similar design (Best et al. 2010; Koelewijn et al. 2014).

**TABLE 2.** Average performance, subjective ratings, and cardiovascular data with SEs in brackets

	Agent		Nonagent Control	
	Single Sentence	Dual Sentence	Single Sentence	Dual Sentence
Performance				
Proportion of words correct	0.95 (0.01)	0.68 (0.03)	0.95 (0.01)	0.67 (0.02)
Total words correct	5.91 (0.05)	8.33 (0.30)	5.84 (0.09)	8.30 (0.31)
Subjective rating				
Effort	4.41 (0.30)	7.78 (0.23)	4.26 (0.30)	7.74 (0.28)
Difficulty	3.36 (0.28)	7.50 (0.27)	3.18 (0.28)	7.57 (0.26)
Performance	8.55 (0.19)	5.55 (0.25)	8.64 (0.15)	5.37 (0.29)
Engagement	6.95 (0.56)	7.15 (0.41)	6.54 (0.55)	6.84 (0.46)
Cardiovascular baseline data				
HF-HRV (n.u.)	44.72 (2.99)	45.06 (3.85)	49.30 (3.90)	45.94 (3.38)
PEP (msec)	103.32 (2.26)	103.60 (2.17)	103.12 (2.26)	103.72 (2.22)
Heart rate (bpm)	69.75 (1.68)	70.43 (1.90)	68.55 (1.81)	69.32 (1.81)
SBP (mm Hg)	118.31 (2.19)	118.54 (1.99)	117.96 (1.88)	118.54 (2.03)
DBP (mm Hg)	73.19 (1.30)	73.88 (1.25)	73.46 (1.26)	72.81 (1.16)
MAP (mm Hg)	82.92 (1.39)	83.12 (1.34)	83.58 (1.28)	83.08 (1.27)
Cardiovascular reactivity data				
HF-HRV (n.u.)	−8.35 (3.16)	−10.59 (2.68)	−8.67 (3.39)	−17.24 (2.63)
PEP (msec)	0.19 (0.45)	0.30 (0.57)	0.60 (0.53)	0.82 (0.54)
Heart rate (bpm)	2.85 (0.53)	2.55 (0.63)	3.71 (0.42)	5.00 (0.72)
SBP (mm Hg)	4.50 (1.41)	5.23 (0.95)	5.50 (0.82)	6.08 (1.02)
DBP (mm Hg)	3.35 (0.49)	3.19 (0.46)	3.38 (0.54)	4.50 (0.82)
MAP (mm Hg)	3.50 (0.49)	3.81 (0.56)	2.54 (0.39)	4.46 (0.83)

Subjective rating scales ranged from 1 to 10, with one decimal precision. PEP reactivity values are expected to be negative, suggesting more effort investment during the task compared with the baseline, however, the opposite was true here. This should be interpreted cautiously however, as the values are very small (all below 1 msec) and the SE values overlap zero in some cases.

DBP, diastolic blood pressure; HF-HRV, high frequency heart rate variability; MAP, mean arterial pressure; PEP, pre-ejection period; SBP, systolic blood pressure.

### Subjective Ratings and Questionnaires

After each block, participants rated their perceived effort investment, the difficulty of the task, their performance, and engagement in the task. The means and SEs for each rating scale are presented in Table 2. Repeated measures ANOVAs (results displayed in Table 3) demonstrated that subjective effort investment and the difficulty of the task increased, while subjective performance decreased, in the dual-sentence compared with single-sentence condition. The VR manipulation did not have a significant effect on any of the subjective ratings. In addition, there were no significant effects of the task demand condition, VR manipulation, nor any interaction between the two on the participants’ reported level of engagement.

**TABLE 3.** Results of repeated measures ANOVAs for subjective rating data

	Effect	*F*(1,24)	*p*	*η*_p_²
Effort	Task demand	**238.53**	**<0.001**	**0.91**
	VR manipulation	0.28	0.60	0.01
	Interaction	0.10	0.76	0.00
Difficulty	Task demand	**214.52**	**<0.001**	**0.90**
	VR manipulation	0.16	0.69	0.00
	Interaction	0.66	0.43	0.03
Performance	Task demand	**310.02**	**<0.001**	**0.93**
	VR manipulation	0.08	0.78	0.00
	Interaction	0.57	0.46	0.02
Engagement	Task demand	0.79	0.38	0.03
	VR manipulation	4.02	0.06	0.14
	Interaction	0.14	0.71	0.00

Significant effects are denoted by bold font.

ANOVA, analysis of variance.

**Fig. 5.**
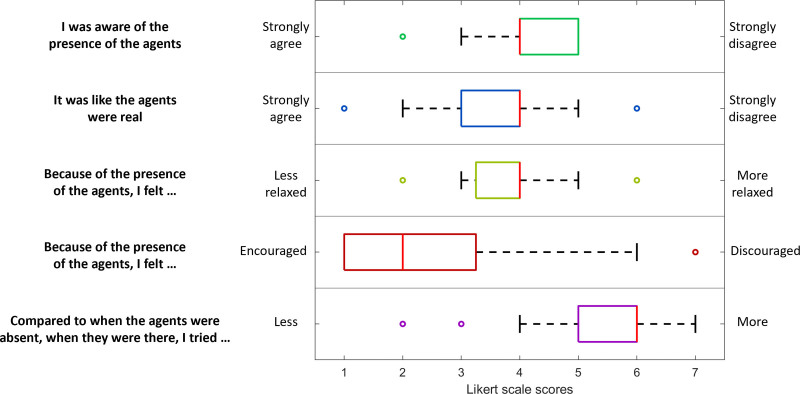
Boxplot demonstrating the spread of answers for the agent questionnaire.

When all blocks of the dichotic listening task had been completed, participants completed the two questionnaires. The results of the i-group presence questionnaire were summed for each participant. The mean score across participants was 41.36 (SD = 5.45). Boxplots of the agent questionnaire results are demonstrated in Figure 5.

### Block-Wise Cardiovascular Analyses

All 26 participants were included in the cardiovascular analyses, except for two with unreliable ICG signals, precluding them from inclusion in the PEP analysis. Average cardiovascular baseline values and SEs for each measure are presented in Table 2. In studies of cardiovascular reactivity, the baseline data are often analyzed to determine any effects of the experimental manipulations on the baseline periods (Richter et al. 2008; Richter & Gendolla 2009). This was deemed particularly necessary here as the agents and nonagent controls were present during the baselines. Despite this, no significant effects were demonstrated in the baseline data (all corrected *p* values >0.05).

**Fig. 6.**
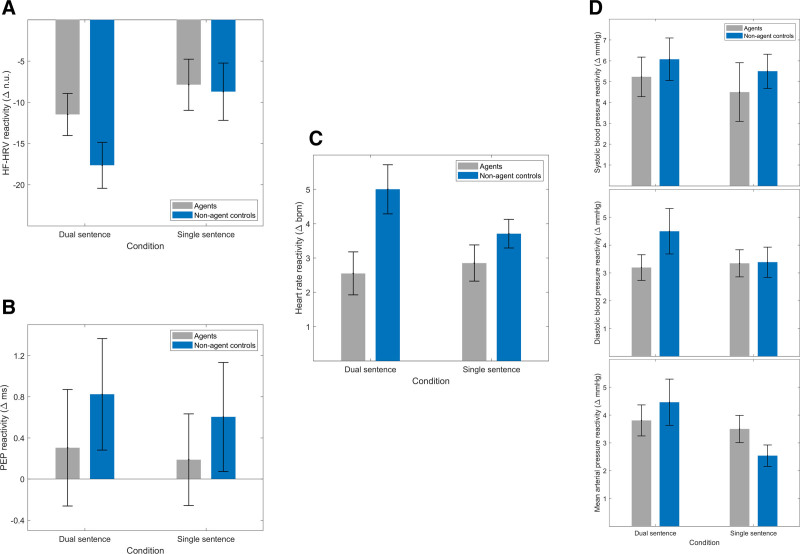
Average cardiovascular reactivity scores with SE bars. (A) HF-HRV, (B) PEP, (C) heart rate, and (D) blood pressure: systolic blood pressure (upper), diastolic blood pressure (middle) and mean arterial pressure (lower). bpm indicates beats per minute; HF-HRV, high-frequency heart rate variability; mm Hg, millimeters of mercury; msec, milliseconds; n.u., normalized units; PEP, pre-ejection period.

Cardiovascular reactivity data are presented in Table 2 and Figure 6. Pearson correlation coefficients revealed that no cardiovascular variables were correlated significantly with BMI, therefore BMI was not included or accounted for in the analysis. Repeated measures ANOVAs were conducted on the reactivity scores to determine the presence of any effects of the VR manipulation or task demand condition on HF-HRV, PEP, heart rate, SBP, DBP, and MAP. Results of this analysis are presented in Table 4. A significant effect of the VR manipulation was demonstrated on heart rate, such that heart rate was higher in the nonagent control conditions compared with the agent conditions. Otherwise, no significant effects were demonstrated on any cardiovascular variables.

**TABLE 4.** Results of repeated measures ANOVAs for cardiovascular measures

	Effect	*F*(1,25)	*p*	*η*_p_²
HF-HRV	Task demand	3.83	0.36	0.13
	VR manipulation	1.84	0.41	0.07
	Interaction	2.08	0.41	0.08
PEP	Task demand	0.23	0.77	0.01
	VR manipulation	3.29	0.36	0.13
	Interaction	0.01	0.94	0.00
Heart rate	Task demand	1.47	0.41	0.06
	VR manipulation	**25.04**	**<0.001**	**0.50**
	Interaction	2.70	0.40	0.10
SBP	Task demand	0.35	0.72	0.01
	VR manipulation	1.41	0.41	0.05
	Interaction	0.01	0.94	0.00
DBP	Task demand	1.12	0.45	0.04
	VR manipulation	1.38	0.41	0.05
	Interaction	0.95	0.47	0.04
MAP	Task demand	3.96	0.36	0.14
	VR manipulation	0.08	0.88	0.00
	Interaction	1.91	0.41	0.07

Significant effects are denoted by bold font. Corrected *p* values are presented. Please note degrees of freedom for PEP analysis were (1,23) due to missing data from two participants.

DBP, diastolic blood pressure; HF-HRV, high frequency heart rate variability; MAP, mean arterial pressure; PEP, pre-ejection period; SBP, systolic blood pressure.

### Exploratory Trial Level Cardiovascular Analyses

**Fig. 7.**
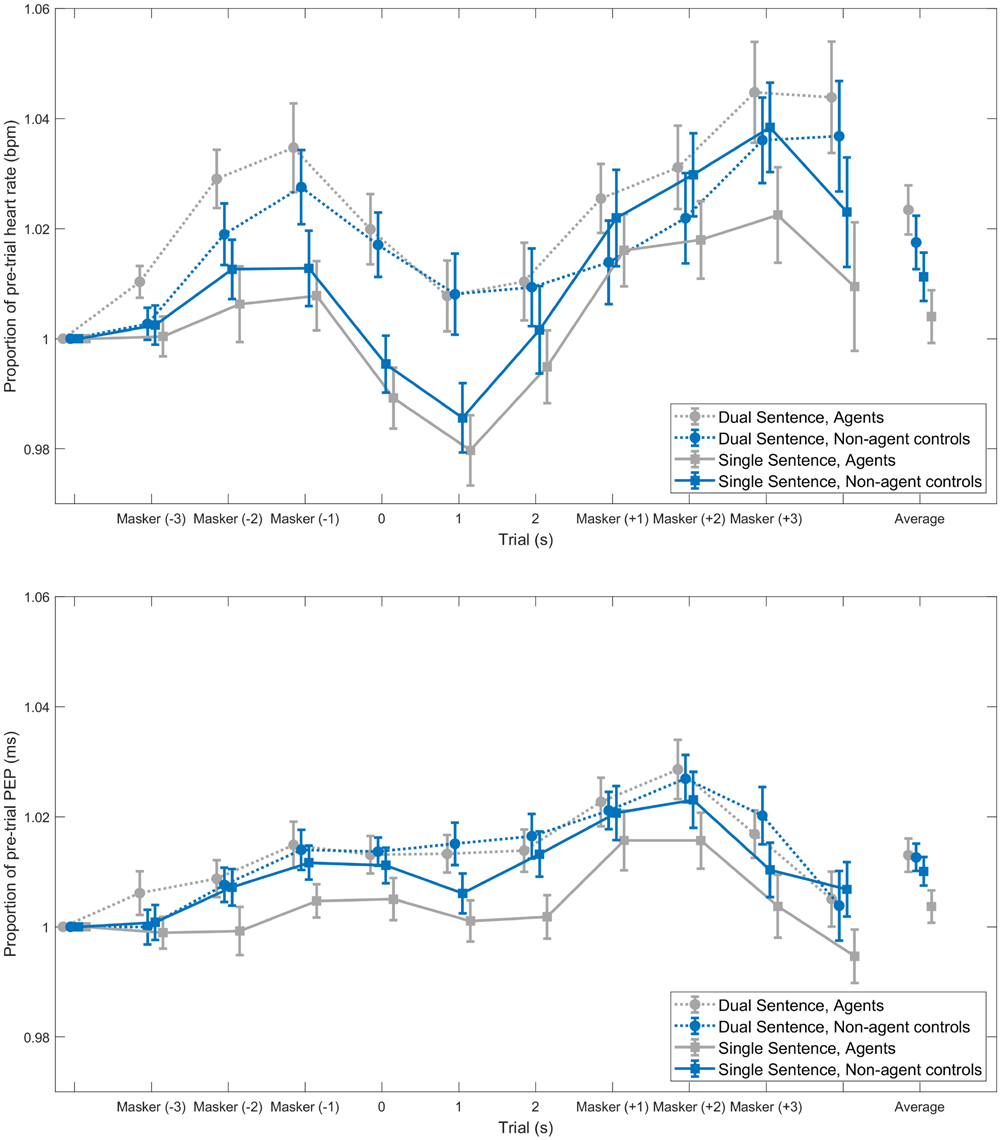
Average heart rate (upper panel) and PEP (lower panel) in 1-sec intervals, presented as a proportion of the value recorded in the silence preceding the onset of the presentence masking noise. Error bars reflect the SE of the mean. bpm indicates beats per minute; msec, milliseconds; PEP, pre-ejection period.

Figure 7 reveals the pattern of average heart rate and PEP over the trials in each of the different conditions. The area under the curves of heart rate and PEP in the three different time windows (presentence masking, dichotic sentence presentation, and postsentence masking) are presented in Table 5. Repeated measures ANOVAs were conducted to demonstrate any effects of the VR manipulation or dichotic listening on these measures within each time interval. Results of this analysis are demonstrated in Table 6. A significant effect of the task demand condition on heart rate was demonstrated in the presentence masking noise [*F*(1,23) = 12.16, *p* = 0.02, *η*_p_² = 0.35] and dichotic sentence presentation phases [*F*(1,23) = 8.62, *p* = 0.03, *η*_p_² = 0.27]. This reveals that in these two-time windows, there was a greater area under the curve (i.e., heart rate was higher) in the dual-sentence condition, compared with the single-sentence condition. Otherwise, no significant effects were demonstrated in any time window, on heart rate or PEP.

**TABLE 5.** Area under the curve averages and SEs

	Agent		Nonagent Control	
	Single Sentence	Dual Sentence	Single Sentence	Dual Sentence
Heart rate				
Masking only	6.03 (0.04)	6.16 (0.03)	6.07 (0.03)	6.11 (0.03)
Sentence presentation	5.91 (0.04)	6.07 (0.04)	5.96 (0.04)	6.07 (0.04)
Postsentence masking	6.11 (0.04)	6.19 (0.04)	6.19 (0.04)	6.14 (0.05)
PEP				
Masking only	6.01 (0.02)	6.05 (0.02)	6.03 (0.02)	6.04 (0.02)
Sentence presentation	6.03 (0.02)	6.08 (0.02)	6.05 (0.03)	6.09 (0.02)
Postsentence masking	6.09 (0.03)	6.14 (0.03)	6.10 (0.03)	6.14 (0.02)

PEP, pre-ejection period.

**TABLE 6.** Area under the curve repeated measures ANOVA analysis

Measure	Time Window	Effect	*F*(1,23)	*p*	*η*_p_²
HR	Presentence masking	Task demand	**12.16**	**0.02**	**0.35**
		VR manipulation	0.13	0.72	0.00
		Interaction	3.24	0.19	0.12
	Sentence presentation	Task demand	**8.62**	**0.03**	**0.27**
		VR manipulation	1.05	0.57	0.04
		Interaction	0.42	0.71	0.02
	Postsentence masking	Task demand	0.24	0.71	0.01
		VR manipulation	0.29	0.71	0.01
		Interaction	3.79	0.19	0.14
Measure	Time Window	Effect	*F*(1,21)	*p*	*η*_p_²
PEP	Presentence masking	Task demand	0.94	0.52	0.04
		VR manipulation	0.01	0.94	0.00
		Interaction	1.21	0.52	0.05
	Sentence presentation	Task demand	4.30	0.27	0.17
		VR manipulation	1.47	0.52	0.07
		Interaction	0.02	0.94	0.00
	Postsentence masking	Task demand	4.00	0.27	0.16
		VR manipulation	0.98	0.52	0.05
		Interaction	0.05	0.94	0.00

Significant effects are denoted by bold font. Corrected *p* values are presented.

ANOVA, analysis of variance; HR, heart rate; PEP, pre-ejection period.

## DISCUSSION

The present study investigated the impact of incorporating VR agents in a dichotic speech reception task. Outcome measures included task performance, subjective ratings (effort, task difficulty, performance, and engagement), and cardiovascular measures. We used two different methods of cardiovascular analysis: a block-wise analysis, where HF-HRV, PEP, heart rate, and blood pressure were computed across complete task blocks, and an exploratory trial-level investigation into heart rate and PEP. We hypothesized that more effort would be invested in the dual compared with the single-sentence condition, causing increased cardiovascular reactivity. In addition, we expected the presence of and evaluation by the agents to increase success importance, and therefore increase the cardiovascular response, compared with the nonagent control conditions.

### VR Agents and Nonagent Controls

The main finding of the block-wise cardiovascular analysis was an effect of the VR manipulation on heart rate. However, the direction of this effect contradicted our expectations. Heart rate was higher in the nonagent control conditions compared with the conditions with agents present. This finding occurred in isolation: it was not accompanied by similar changes in performance, subjective ratings, or any other cardiovascular measures (HF-HRV, PEP, or blood pressure measures).

There are two primary aspects to discuss regarding these cardiovascular findings. The first is the lack of evidence for the expected effect (i.e., why was there no measurable decrease in HF-HRV or increase in PEP, heart rate, SBP, DBP, and MAP in the presence of the agents?) and the second is the presence of the opposite effect (i.e., why did heart rate increase more in the absence of the agents?). A potential reason for the former may be due to the realism of the agents. Around half (46%) of the 24 participants who provided additional comments in the free written part of the agent questionnaire explicitly commented in the free written part of the agent questionnaire that the agents were not realistic in their movements, interaction, or facial expressions. Some examples of comments on the agent questionnaire item 2 (“It was like the agents were real”), translated from Dutch to English include: “they did not resemble real humans and I knew it was a VR world,” “I could clearly notice that they were not real,” “they were moving too robotically” and “mainly the faces were unrealistic.”

A lack of realism may have detracted from the participants’ feeling of presence in the virtual environment (Baños et al. 2005; Diemer et al. 2015). This is supported by the i-group presence questionnaire results, which were lower than have been demonstrated in other studies (Vasconcelos-Raposo et al. 2016; Hruby et al. 2020). To increase presence, studies using VR versions of the TSST have included evaluators who speak (Shiban et al. 2016), write notes on the paper in front of them (Standard et al. 2020), move their gaze and reposition their feet (Liu & Zhang 2020). One study even incorporated real-time eye tracking of the participant, to allow eye contact between the participant and virtual audience members (Zimmer et al. 2019). More realistic animations in the present study may have been more convincingly elicited a sense of “presence” for the participants.

Another potential reason for a lack of evidence for the expected effects in the cardiovascular measures relates to the timings of the stimuli presented in this study. The proportion of each trial dedicated to listening to the presented sentence was small: just 1.84 sec on average, compared with 6 sec of masking noise (3 sec before and after the dichotic sentence presentation), the participant response time, scoring time and finally, 2 sec of agent feedback. The additional nonsentence presentation time during each trial may have added additional noise to the cardiovascular signals, obscuring any true response. Supporting this theory, a previous study demonstrated that PEP was more sensitive to changes in task demand when extracted during the dichotic sentence presentation of a speech-in-noise trial, rather than averaged across whole task blocks (Plain et al. 2020).

The second point for discussion is that the heart rate response was larger in the nonagent control conditions compared with the agent conditions, or phrased in the opposite way, the heart rate response was smaller with the agents present. This finding is surprising as generally social presence (physical and virtual) is associated with either increased or unchanged heart rate (Wright et al. 1998; Gendolla & Richter 2006; Fairclough & Roberts 2011; Plain et al. 2021). For example, in studies incorporating a physical social presence, heart rate increased while participants were evaluated (Wright et al. 1998) or given performance feedback (Fairclough & Roberts 2011), yet remained unaffected by observation (Gendolla & Richter 2006; Plain et al. 2021). Similarly, some studies incorporating virtual social agents in the TSST revealed increases in heart rate (Shiban et al. 2016; Zimmer et al. 2018; Fallon et al. 2021), though the response was smaller than that elicited by the in person TSST.

Here, a smaller response with the agents present could be attributed to a supportive or reassuring effect of the evaluation or feedback provided by the agents. In the nonagent control condition, participants received no performance feedback, whereas in the agent condition, they were shown occasional positive or negative animations reflecting whether they were correct or incorrect. Positive feedback was given more often than negative feedback: up to five positive animations and three negative animations were shown per block of 30 sentences. The feedback may have provided reassurance to the participant that they were succeeding at the task. Indeed, the results of the agent questionnaire (Fig. 5) suggest that generally the presence of the agents was encouraging, though there was a significant spread across the Likert Scale. Some studies suggest that social support reduces heart rate (Vrana & Rollock 1998; Ditzen et al. 2007), though it should be noted that these studies included physical touch, which is not directly relevant here.

Another option is that the participants may have simply been more distracted by the animations of the agents with feedback than the nonagent controls (i.e., the measured effect may have been unrelated to social-evaluative threat and purely due to a calming visual distraction). Indeed, during some medical or dental procedures, visual distraction in VR is used as a tool to reduce anxiety levels (Van Twillert et al. 2007; Kaur et al. 2021; Bernaerts et al. 2022). It is interesting that there was no measurable difference in performance between the agents and nonagent control conditions, suggesting that participants were not distracted sufficiently to affect their performance level.

Alternatively, a greater physiological response to the nonagent controls may have resulted from their somewhat uncanny resemblance to humans, which may have triggered evaluative threat. Future work would benefit from the separate manipulation of visual distraction, VR agents, and feedback, to allow more definitive conclusions to be made about the origins of any physiological changes seen. Future work may also benefit from the inclusion of more in-depth subjective rating items, providing a deeper understanding of participants’ perception of the agents. For example, other authors have included items specifically related to copresence, embarrassment, and likeability (Bailenson et al. 2005).

### Dichotic Listening Conditions

In keeping with the results of other dichotic speech reception studies, performance was poorer in the dual-sentence condition, compared with the single-sentence condition (Best et al. 2010; Koelewijn et al. 2018). In the present study, stimuli were presented at −3 dB, matching one of the conditions applied by Koelewijn et al. (2014). At this SNR, Koelewijn et al.’s participants obtained average performance scores of 62% for the dual-sentence condition and 89% for the single-sentence condition, not dissimilar from the performance measured here (around 68% and 95%, respectively). Subjective ratings were also sensitive to the dichotic listening condition: participants recognized that their performance decreased, while their subjective effort investment and perceived task difficulty increased, in the dual-sentence condition compared with the single-sentence condition.

Surprisingly, none of the block-wise cardiovascular measures were affected by the task demand manipulation. In particular, we expected to see a larger decrease in HF-HRV (revealing more effort) in the dual-sentence condition compared with the single-sentence condition, as this measure has been shown to be sensitive to task complexity during a dichotic digit listening task, which also involved dividing attention between the two ears (Seeman & Sims 2015). Notably, Seeman and Sims (2015) measured changes to HRV in response to their dichotic manipulation, even when performance was near ceiling (above 97%) for all conditions. As described earlier, performance in the present study was lower in all conditions, suggesting that the task required effort investment. It could also be argued that the task was more difficult in the present study, because participants were required to repeat whole sentences, rather than digits. In addition to different tasks, that is, digits versus sentence presentation, the dissimilar cardiovascular results between Seeman and Sims’ and the present study could be attributed to differences in measures. We calculated the high-frequency component of the HRV (HF-HRV), which is thought to reflect parasympathetic nervous system activity, whereas Seeman and Sims presented the SD of normal-to-normal intervals, which is a more mixed autonomic measure (Malik et al. 1996). It is interesting that Mackersie and Cones’ (2011) participants underwent a similar dichotic digit task, with near-ceiling level performance in their different conditions, but no changes in heart rate were revealed.

### Trial Level Analysis

#### Heart Rate

One of the additional aims of this study was to explore patterns of heart rate and PEP within a trial. To achieve this, we conducted an analysis similar to that of Francis et al. (2016), which involved averaging the measures (here, heart rate, and PEP) in 1-sec interval during the trial and normalizing them to pretrial values. The timings of stimuli presented by Francis et al. differed from those in our study. Francis et al.’s trials began with 0.5 sec of silence, followed by the target sentence (2.8 to 3.5 sec), then 8 sec of silence, before the participants responded. In our study, 3 sec of masking noise preceded and followed the target sentences, and the participant responded immediately after this, without any enforced periods of silence. Then, after the participant’s verbal response, the experimenter scored the response, and there was a 2-sec interval for any performance feedback to be given by the agents.

Despite these timing differences, there are some similarities between the patterns of heart rate in the studies. For example, both studies reveal a slight increase in heart rate after the onset of the trial (after sentence onset for Francis et al. [2016], and the masking onset in our study), followed by a subsequent decrease in heart rate. In Francis et al.’s (2016) results, the dip in heart rate occurs at around 7 sec after the sentence onset, whereas this occurs sooner in our study, around 5 sec after masking onset or during the dichotic sentence presentation. A decrease (i.e., slowing) in heart rate is reported in the phasic heart rate literature to reflect anticipation of the response (Jennings & Van Der Molen 2002). We suggest that in our data, heart rate slows as the participants are preparing for the sentence presentation to begin.

To test our original hypotheses on the trial level heart rate and PEP data, we conducted repeated measures ANOVAs on the areas under the curves in three different time windows: the presentence masking, dichotic sentence presentation, and the postsentence masking. This analysis revealed that during the first two of the three-time windows, there was a significant effect of the task demand on heart rate, demonstrating that the area under the curve was greater (i.e., heart rate increased) in the dual-sentence condition compared with the single-sentence condition. This is of particular interest because acoustically the dual- and single-sentence conditions were identical, which means that the changes demonstrated are not simply due to the level of the sound, as has been shown in other studies (Shoushtarian et al. 2019). The difference between heart rate in the dual- and single-sentence trials in these windows is likely to be caused by the knowledge and anticipation that the dual-sentence trials will be more difficult, compared with the single-sentence condition.

The higher heart rate during the dual-sentence condition compared with the single-sentence condition is then maintained during the presentation of the stimulus but disappears during the latter part of the trial (postsentence masking). This may reflect that the data selected for this part of the analysis was contaminated with other parts of the trial, or even the participant response time. For example, the windows for analysis were always split into 3-sec intervals (3-sec presentence masking, 3-sec dichotic sentence presentation, and 3-sec postsentence masking), regardless of the sentence duration. If sentence presentation was brief in one trial, the data selected for analysis as the postsentence masking noise, may actually reflect mainly the participant response. The lack of a fixed trial duration and an interval of silence before the participant response, are limitations of the present analysis.

The area under the curve heart rate analysis revealed no significant effect of the VR manipulation. This is likely to relate to the timings of the trial, specifically with regards to the feedback timing. Several studies have revealed that providing performance feedback results in heart rate changes that occur almost instantly, affecting two to three interbeat intervals after the feedback is presented (Somsen et al. 2000; Crone et al. 2003; Van Der Veen et al. 2004). In the present study, the trial level heart rate data were limited to the period before the feedback was given (i.e., the presentation of the presentence and postsentence masking noise and the dichotic sentence presentation). The timing of the feedback presentation is not precisely known, due to unfixed response and scoring durations. It is therefore possible that the feedback caused a short-lived response that was not captured during the trial-level analysis window. Future work would benefit from more optimized, fixed trial structure to capture responses to the feedback. In addition, it may prove informative in future work to explore the responses to different types of feedback (positive, negative, or no feedback trials). Negative feedback, for example, has been shown to cause a slowing of heart rate (Somsen et al. 2000; Jennings & Van Der Molen 2002; Van Der Veen et al. 2004).

#### Pre-Ejection Period

In contrast with heart rate, no significant trial level fluctuations in PEP were elicited by any of the task manipulations, in any time window. This could relate to the timings of stimulus presentation (the relatively short period of sentence presentation in each trial, as discussed earlier) but also the autonomic nervous system origins of PEP. Changes in PEP result from sympathetic nervous system activity on the heart, the influence of which is known to act slower than the parasympathetic nervous system (Draghici & Taylor 2016; Christensen et al. 2020). It is possible that any changes elicited in PEP at the trial level appeared after the time window selected for analysis or impacted the subsequent sentence (and thus the baseline correction of the next sentence).

An additional contributory factor, and a limitation of the present study, may relate to the semiautomated procedure used in the PEP analysis. We devised our own novel algorithm to detect the relevant features of the ECG (Q-point) and ICG (B-point) signals. Though care was taken to visually inspect the output of our algorithm, it is well known that the B-point of the ICG especially can be very difficult to determine accurately in an automated fashion (Sherwood et al. 1990). This is due to signal artifacts caused by body movements and respiration, as well as inherent variability in the morphology of the signal between individuals (Forouzanfar et al. 2018). Several automated procedures for determining PEP have been suggested (Szilágyi et al. 1992; Berntson et al. 2004; Lozano et al. 2007; Arzeno et al. 2008; Árbol et al. 2017), but they do not perform optimally, and therefore expert visual inspection performed by multiple scorers (as was conducted for the block-wise analysis in this study) is the recommended approach (Sherwood et al. 1990; van Lien et al. 2013). The semiautomated tangential method used in this study to detect the B-point differs from other approaches applied in the literature (see examples of some B-point detection algorithms here: Forouzanfar et al. 2018). It was beyond the scope of this work to validate the algorithm itself. Thus, the results of the trial-level PEP analysis should be interpreted with caution.

An additional limitation affecting the trial level analysis is that the precise onset and duration of each blood pressure measurement were not recorded, meaning that trials occurring during blood pressure measurement were included in the analysis. This is a limitation because the blood pressure cuff inflation itself may have had an impact on the participant during the affected trials. For instance, some individuals find blood pressure cuff inflation uncomfortable (Del-Río-Guerrero et al. 2023). This impact was not anticipated to be substantial, however future trial-level work may benefit from exclusion of blood pressure-contaminated trials.

## CONCLUSIONS

This study measured the cardiovascular reactivity of normally hearing individuals during a dichotic speech reception task in two VR conditions: in the presence of agents who provided performance feedback, or in the presence of nonagent controls, without any performance feedback. Contrary to our expectations, an increase in heart rate was demonstrated during the nonagent control conditions, compared with the agent conditions. This was not reflected in any other cardiovascular measure. In contrast, performance and subjective ratings were sensitive only to the dichotic condition: that is, dual-sentence or single-sentence condition. An exploratory analysis revealed that heart rate fluctuations within a trial differed between the dichotic conditions: heart rate was higher in the first two-thirds of the trial when two sentences had to be repeated, compared with just one sentence. This analysis demonstrates that trial-level cardiovascular measures can be successfully extracted, and analysis of these shorter segments may contain different and supplementary information to that obtained when data are averaged across a block.

## ACKNOWLEDGMENTS

The authors thank J. H. M. van Beek for his support in experimental setup.

## Footnotes

* The two repeated condition orders were as follows: (1) dual sentence/nonagent controls, (2) single sentence/agents, (3) dual sentence/agents, (4) single-sentence/nonagent controls; and (1) dual sentence/nonagent controls, (2) single-sentence/ nonagent controls, (3) dual sentence/agents, (4) single-sentence/agents.
